# Supplementary material for: Digital Marketing of Commercial Complementary Foods in Australia: An Analysis of Brand Messaging
Source: Int J Environ Res Public Health. 2021 Jul 27;18(15):7934. doi: 10.3390/ijerph18157934 (PMC8345376; doi:10.3390/ijerph18157934)
Supplement: Supplementary file 1 [file ijerph-18-07934-s001.zip › ijerph-1283413-SI.pdf]

International Journal of
Environmental Research and Public Health

Supplementary Tables S1–S3

Table S1. Definitions of code categories used to analyze commercial complementary food brands Facebook post messages.

Message Coding Category	Text or image referring to…
Taste/Flavor	any element of taste (yummy/tasty etc.) or flavor of product (apple/carrot etc.) and flavor variations (have you tried *x*/*y* flavor)
Texture	product texture (crunchy/smooth etc.) or reference to texture exposure (new textures for baby to explore)
New	new product or flavor, including new product teasers (e.g., coming soon)
Organic	organic status (including certification) of product or ingredients
Additive/Allergen Free	product or ingredients that are free from known allergens or additives such as artificial colors/flavors/preservatives, GMOs as well as added sugar or salt.
Referring to Child Age	age recommendations for feeding (suitable from 6 months/12+ m etc.) or specific age stage (fussy toddler/busy preschooler etc.), includes pack shot where age is prominent on deliberately focused package only.
Appropriate for <6 months	age recommendations that indicate product is suitable for infants under 6 months of age (e.g., 4 months +) or statements that indicate feeding may be initiated before 6 months of age (e.g., suitable for babies from 5 months)
Self – Feeding	promoting child ability to self-feed (finger food, baby led weaning etc.) by product suitability for such or importance of this skill
Fun/Positive Experience	creating a happy or fun experience when eating (positive food experience/mealtime adventures/exploring fun foods/mealtime playtime etc.)
Solution to a Problem	product or brand offering a solution to a (real or perceived) issue for parents (on the go snack/fussy eater approved/no mess)
Child Development/Growth	product or ingredient effect on growth or aid to development (helps chewing skills/grow strong bodies etc.)
Health Support/Healthy Habit	supporting the development of "healthy habits" (e.g., develop a good relationship with food) or health aspect (immune function/easy to digest etc.)
Healthy Ingredients/Nutrition	ingredients commonly known to be healthy (fruit/vegetables/grains etc.) or referencing the 'nutritious' aspects (includes buzzwords like 'goodness'/'wholesome') or specific nutrients (iron/vitamin C etc.)
Milks	toddler/growing up milks or other formula like products
Stockists	retailers or locations products are stocked
Refer/link to website	direct viewers to the brands website (recipe/tip links)
Australian	ingredients, suppliers or company being Australian/locally owned or made
Images of child	positive (happy/cute/funny/sweet) images of babies, toddlers and children, double tagged (++) if product package is clearly shown in also.
Other	aspects that promote the brand/products that do not fit with described categories. Describe briefly in column

Table S2: Definitions of categories used to assess commercial complementary food brands Facebook post aims.

Facebook Post Category	Post is broadly …

Product Range	intending to promote a specific product or product line (e.g., multiple flavors of one product type), including links to recipes using product
Broad Values	intending to promote multiple product ranges, ingredients or company values in general; includes cover picture updates
Incentives	a competition or giveaway (includes terms and conditions posts)
Social/Parenting Support	offering parenting advice or support without specific product mention (may be pictured) or expert/celebrity mentioned
Engagement/Connection Building	attempting to build an emotional connection through humor, shared experience, invitation to post pictures (not related to incentives); includes polls, charitable work and "well wishing"
Expert/Celebrity Endorsement	showcasing celebrity or health professional (expert) involvement with the brand or advice from such
Informative	offering other company information not related to product range/broad values/parenting support

Table S3. Examples of messages and products promoted by commercial complementary food brands in Facebook posts by coding attribute.

**Attribute**	**Message**	**Product (Brand); Food Category**
**Theme 1 –General Product Attributes**		
**Taste/Flavor**	"The number 1 most delicious snack for little ones!"	Veggie Straws (Kiddylicious); Ready-to-eat Snack
	"A tasty way to get goodness"	Sweet Potato and Apple Lentil Sticks (Whole Kids); Ready-to-eat Snack
	"…the perfect snack to introduce your little one to exciting new textures and flavors!"	Snack Bars, various flavors (Rafferty's Garden); Ready-to-eat Snack
**Texture**	"… deliciously creamy…"	Chicken, cheddar cheese, cauliflower and rice Squeeze Pouch (Only Organic); Ready-to-eat Meal
	"With a crunchy outside and tasty veggie filling…"	Tomato and Basil, Carrot Straws (Rafferty's Garden); Ready-to-eat Snack
**New product**	"These new products will be an essential addition to both baby and parent feeding journeys"	Smoothie Bowls (Bellies); Ready-to-eat Meal
	"Our new Puffs snacks will be arriving in supermarkets soon, so we thought we'd give our Facebook fans the chance to be the first to give them a try!"	Strawberry and Apple, Mango and Banana Puffs (Only Organic); Ready-to-eat Snack
**Theme 2 – Socially Desirable Attributes**		
**Self - Feeding**	"Strawberry Fruity Puffs are not only delicious and great for baby led weaning but they are also made with real fruit"	Strawberry Fruity Puffs (Kiddylicious); Ready-to-eat Snack
	"They're also the perfect size and shape for little hands and mouths"	Strawberry and Apple, Mango and Banana Puffs (Only Organic); Ready-to-eat Snack

**Fun/Positive Experience**	"The best way to get your baby engaging with food is by making snack time fun!"	Round-a-bouts (Bellies); Ready-to-eat Snack
	"How can you make mealtime more meaningful? Getting your kids involved whilst preparing a meal helps develop the ability to think about eating in a more mindful manner"	No product (Bellies)
	"…the perfect snack for fuelling little adventures"	Tomato and Basil, Carrot Straws (Rafferty's Garden); Ready-to-eat Snack
**Solution to a Problem**	"Fussy eaters in the family? Pasta is a great food to use when trying to expand a fussy eaters repertoire. Try our rage of different shapes and textures"	Pasta range (Bellamy's Organic); Ready-to-cook
	"Stuck on meal ides for your little one? Join Nestle Mum and me today for easy and delicious baby recipes they'll love!"	No product (Nestle)
	"…we've got a range of hearty savoury meals that can be ready to serve in minutes for lunch, dinner or anything in between!"	Squeeze pouch range (Only Organic); Ready-to-eat Meal
	"Soothe those teething troubles with our Banana Milk Teething Rusks"	Banana Milk Rusks (Rafferty's Garden); Ready-to-eat Snack
**Australian made/owned**	"Bubs Organic Pouches are proudly Australian Made and Owned"	Squeeze pouch range (Bubs); Ready-to-eat Meal
	"…beautiful organic dairy farm in Inverloch, Victoria. Third generation dairy farmers, their delicious organic milk goes into our yoghurts, custards and toddler milks"	Product range (Bellamy's Organic)
**Theme 3 – Concern-based Attributes**		
**Organic**	"In Australia, there are over 250 different synthetic pesticides and fertilisers used on conventional farms to protect fruits and vegetables from pests"	Whole product range (Bellamy's Organic)
	"Bubs Organic baby food is proudly certified organic by ACO."	No product (Bubs)
**Additive/Allergen Free**	"no artificial colors, no artificial flavors, no artificial preservatives, no GMOs"	Whole product range (Bubs); Ready-to-eat Snack
	"…preservative free…real ingredients….nothing artificial…"	Farm animal biscuits (Whole Kids); Ready-to-eat Snack
**Referring to Child Age**	"We have plenty of age-appropriate wholesome snack options that support all stages of child development – from the earliest motor skills to ongoing positive nutrition"	Whole product range (Bellies); Ready-to-eat Snack

	Try Bubs Organic Baby Cereal suitable from 4months+. For older bubs top with fresh fruit and a sweetener of choice, such as maple syrup"	Cereal (Bubs); Ready-to-prepare
**Theme 4 – Health- focused Attributes**		
**Child Development/Growth**	"Naturally gentle, premium nutrition enriched with 16 essential vitamins and minerals to support your toddler's growth and development"	Goat Toddler Milk (Bubs)
	"They melt easily in the mouth to encourage safe self-feeding and tongue lateralisation. This is and important skill for feeding therapy and development"	Banana Softcorn (Bellies); Ready-to-eat Snack
	"Snacks are a great way to begin developing your baby's motor skills by supporting their pincer grip and coordinating complex food shapes in their mouths; all so important for a developing eater!"	Unspecified snack product (Bellies); Ready-to-eat Snack
**Health Support/Healthy Habit**	"It's never too early to introduce healthy habits"	Apple and Cinnamon Puffs, Banana Softcorn (Bellies); Ready-to-eat Snack
	"Packed full of nutrients to support their immune system"	Organic Grass Fed Toddler Milk (Bub's)
	"Our nutrition contributor @wholefoodhealing setting healthy habits with her daughter Clemmie"	Apple snacks (Bellamy's Organic); Ready-to-eat Snack
**Healthy Ingredients/Nutrition**	"Enriched with vitamins and minerals, such as Iron and Vitamin C, for added nutrition"	Cereal (Bubs); Ready-to-prepare
	"As a guide, offer three small meals, and 2-3 snacks each day. Offer nutritious snacks of fruit, milk products and whole grain cereals"	Apple and Pear Snacks (Bellamy's Organic); Ready-to-eat Snack
	"… our Lentil Puffs have a 5 Star Health Rating"	BBQ, Salt and Vinegar Lentil Puffs (Bellies); Ready-to-eat Snack
	"Dairy free, packed with protein, made with chickpeas"	Houmous Dip-Dip (Kiddylicious); Ready-to-eat Snack
	"Like you, we prepare our savoury meals using freshly chopped organic veggies and pieces of lean organic meat before sealing in the goodness to provide nutritional, tasty meals for your babies and toddlers"	Whole product range (Only Organic); Ready-to-eat Meals
	"Just honest food with real ingredients, created by a real Mum"	Whole product range (Whole Kids); Ready-to-eat Snack
	"Our secure local process helps to deliver fresher product, better nutritional values, better taste and better solubility"	Goat Toddler Milk (Bubs)
